# SHP-1 Regulation of Mast Cell Function in Allergic Inflammation and Anaphylaxis

**DOI:** 10.1371/journal.pone.0055763

**Published:** 2013-02-04

**Authors:** Li Zhou, Sun Young Oh, Yuqi Zhou, Baojun Yuan, Fan Wu, Min Hee Oh, Yefu Wang, Cliff Takemoto, Nico Van Rooijen, Tao Zheng, Zhou Zhu

**Affiliations:** 1 Division of Allergy and Clinical Immunology, Johns Hopkins Asthma and Allergy Center, Baltimore, Maryland, United States of America; 2 Division of Hematology, Department of Pediatrics, Johns Hopkins University School of Medicine, Baltimore, Maryland, United States of America; 3 Center for Animal Experiment/ABSL-III Laboratory and State Key Laboratory of Virology, College of Life Sciences, Wuhan University, Wuhan, P.R. China; 4 Department of Molecular Cell Biology, VU Medical Center, Amsterdam, The Netherlands; Leiden University Medical Center, The Netherlands

## Abstract

Allergic inflammation and severe allergic reactions (anaphylaxis) are important in allergen induced diseases. Bacterial products such as lipopolysaccharide (LPS) are ubiquitous and can facilitate allergen induced Th2 immune responses. Phosphatase SHP-1 is critical in regulating immunological homeostasis and in allergen induced Th2 immune responses in the lung. However, the mechanisms underlying the initiation of allergic inflammation and allergen induced anaphylaxis are still not completely elucidated and it is unclear whether SHP-1 plays any role in LPS-induced airway inflammation and in allergen-induced anaphylaxis. In this study we tested the hypothesis that phosphatase SHP-1 plays an important role in allergic inflammation and anaphylaxis and determined whether its effects are through regulation of mast cell functions. SHP-1 deficient (*mev*/+ and *mev/mev*) and mast cell deficient (*Kit^W-sh^*) mice were examined in their responses to LPS airway stimulation and to ovalbumin (OVA) allergen induced systemic anaphylaxis. Compared to wild type mice, *mev*/+ mice had significantly enhanced LPS induced airway inflammation and OVA induced anaphylactic responses, including hypothermia and clinical symptoms. These changes were mast cell dependent as *Kit^W-sh^* mice had reduced responses whereas adoptive transfer of mast cells restored the responses. However, T and B cells were not involved and macrophages did not play a significant role in LPS induced airway inflammation. Interestingly, basophil differentiation from SHP-1 deficient bone marrow cells was significantly reduced. These findings provided evidence that through regulation of mast cell functions SHP-1 plays a critical role as a negative regulator in allergic inflammation and in allergen induced anaphylaxis. In addition, SHP-1 seems to be required for normal basophil development.

## Introduction

Allergic asthma, food allergy and anaphylaxis, are common disorders with high prevalence in the United States [1], [2], [3]. Abnormal immune responses in susceptible individuals to otherwise innocuous antigens are believed to be responsible for the clinical manifestations. The common pathways in the pathogenesis of allergic diseases involve activation of antigen-specific Th2 cells, production of Th2 cytokines, generation of antigen-specific immunoglobulins, especially IgE, sensitization and upon re-exposure to allergen, activation of mast cells and basophils. However, the mechanisms that control the susceptibility to allergen sensitization and responses are still not well understood, particularly the factors that negatively regulate the processes.

Inflammation is an important component in the pathogenesis of asthma. However, the mechanisms by which inflammation is involved in initiation of asthma and allergy are not clear. Studies have found that clinical manifestations of allergic asthma in young children are inversely correlated with the exposure levels of bacterial product endotoxin or lipopolysaccharide (LPS), thus the "hygiene hypothesis" [4]. However, other studies found that LPS exposure may exacerbate symptoms of asthma [5]. Studies, including our own, in experimental models revealed that LPS demonstrated different modulating effects on specific immune responses to allergens depending on the exposure levels of LPS [6], [7], [8]. However, the signaling pathways, participating cell types, and modulating factors in this process have not been completely elucidated.

Mast cells are important in airway inflammation, asthma, allergy and anaphylaxis. In humans, mast cells are a major effector cell type in allergic responses, particularly anaphylaxis. Mast cell degranulation and mediator release in the airways are associated with airflow obstruction in asthmatic patients [9], [10]. In mouse models, mast cells and associated pro-inflammatory cytokines play an important role in airway inflammation and immune responses to aeroallergens [11], [12].

Phosphatase SHP-1 is an important regulator in various signaling pathways [13], [14]. The major function of SHP-1 is to limit the extent of activation and cellular responses to stimulation by dephophorylating its target molecules. In humans, reduced expression of SHP-1 at mRNA or protein levels has been seen in association with some leukemia and lymphoma cell lines [15], in polycythemia vera and in multiple sclerosis [16], [17]. Furthermore, it has been reported that reduction of SHP-1 expression in multiple sclerosis patients may be caused by virus-induced increased methylation of the SHP-1 promoter [18]. In mice, the biological significance of SHP-1 is highlighted in the severe inflammatory phenotypes of two mutant strains, motheaten and viable motheaten [19], [20], [21], [22]. Studies, including ours, have shown that SHP-1 is a critical negative regulator in the generation of allergic inflammation in the airway and in the lung [23], [24], [25], [26], [27]. More recently, SHP-1 was shown to regulate mast cell differentiation and responses to various stimulations [27].

In this study, by using SHP-1 deficient and mast cell deficient mice in models of LPS induced airway inflammation, IgE-FcεRI mediated passive systemic anaphylaxis (PSA) and OVA allergen induced active systemic anaphylaxis (ASA), we tested the hypothesis that SHP-1 through regulation of mast cell functions plays a critical role in controlling airway inflammation and anaphylaxis.

## Results

### Enhanced tissue-derived mast cell development in SHP-1 deficiency

**Figure 1:**
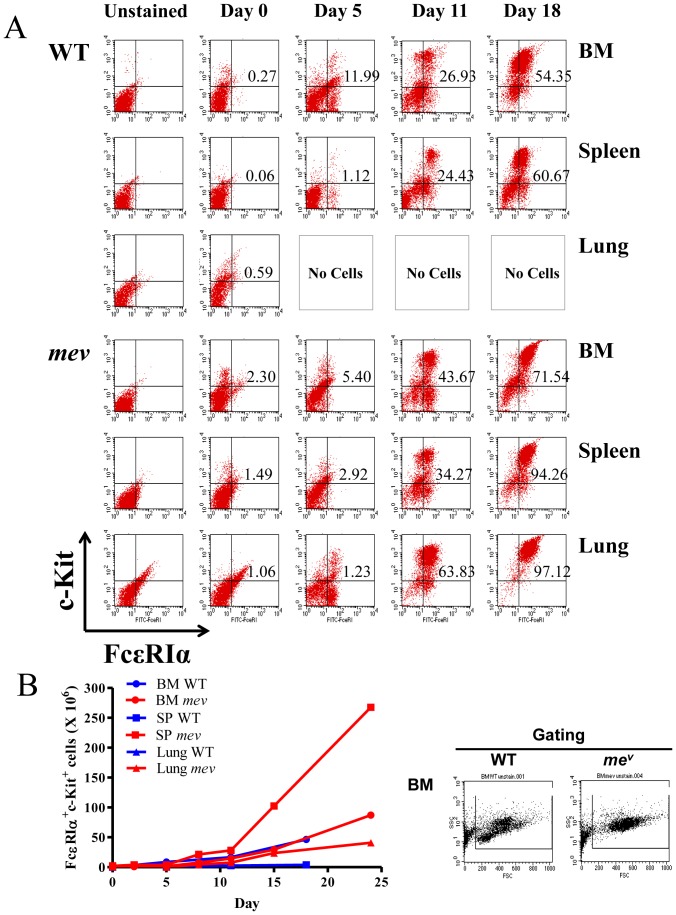
SHP-1 deficiency resulted in enhanced tissue-derived mast cell development. Tissue cells from bone marrow (BM), spleen (SP) and lung were obtained from wild type (WT) and *mev/mev* mice and seeded at 4×10^5^/mL and cultured in IL-3-containing WEHI conditioned medium. (**A)**. At specified time, FcεRI+ and c-Kit+ mature mast cells were determined among gated cells by flow cytometry as described in the Methods. The numbers are the percentage of total cells after gating. No cells from the lung of WT mice survived in culture. (**B**). The total number of mast cells derived from WT and *mev/mev* bone marrow, spleen, and lung at different time points in culture. Shown is a representative of three experiments with similar results.

To better understand SHP-1 regulation of mast cells in tissues, we examined mast cell development in extramedullary tissues of WT and *mev/mev* mice, which was compared with mast cells from bone marrow. Unlike bone marrow, no mast cells could grow from lung tissue of WT mice (**Figure 1A and 1B**). On the other hand, mast cells were readily derived from lung tissue of *mev/mev* mice, although the total cell number was lower than that from bone marrow (**Figure 1A, 1B**). Interestingly, mast cells derived from spleen tissue of both WT and *mev/mev* mice but with different patterns. Mast cells from WT spleen differentiated slowly and the total number of cells was barely above the baseline. In contrast, mast cells derived from *mev/mev* spleen showed a rapid differentiation rate with a total cell number four-fold higher than that of bone marrow-derived mast cells (**Figure 1A, 1B**).

### SHP-1 and Mast Cells in Airway Inflammatory Responses to LPS

**Figure 2:**
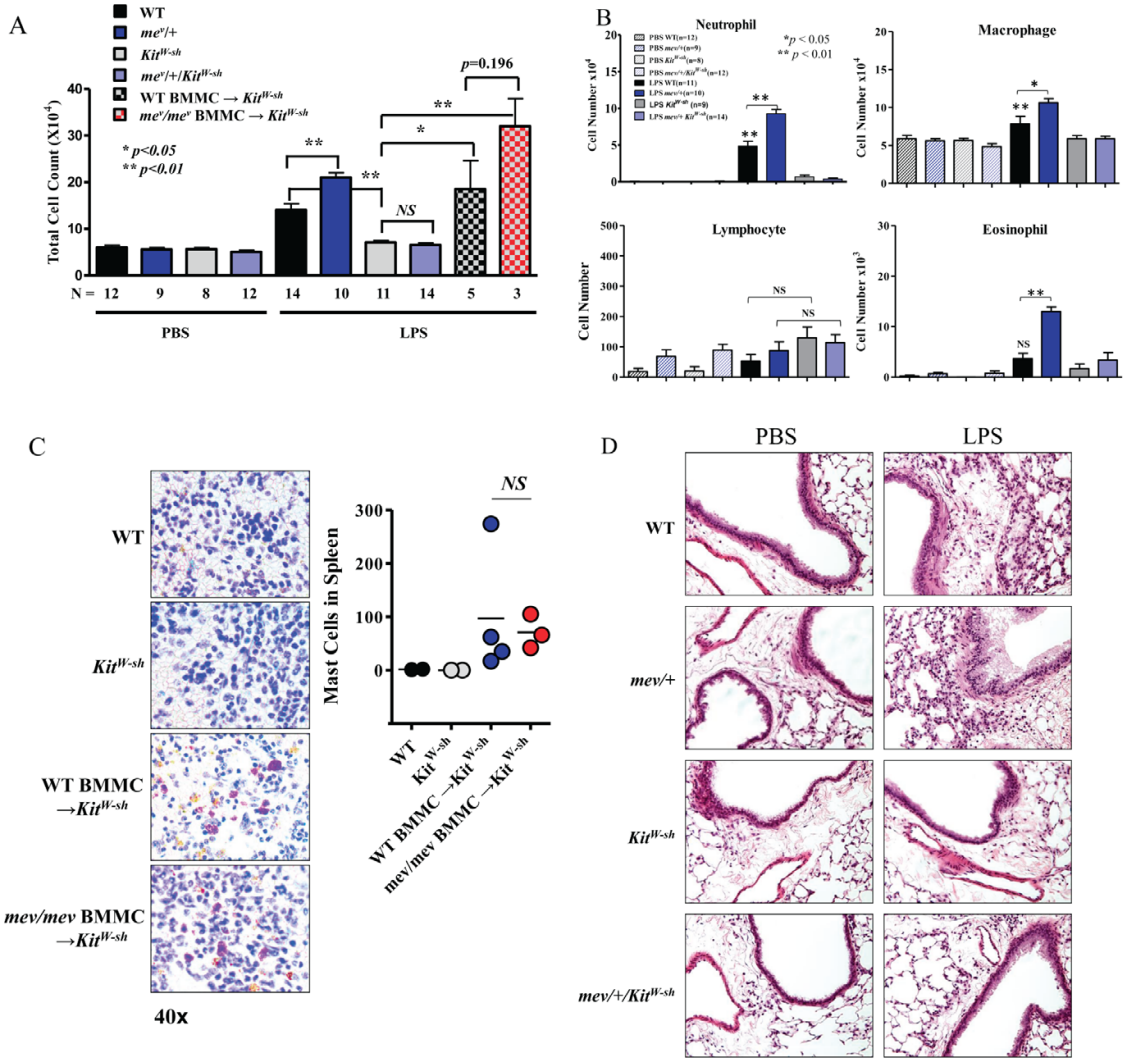
SHP-1 and mast cell regulation of LPS induced airway inflammation. Mice were stimulated with LPS (100 ng) or PBS by intranasal (i.n.) instillation. (**A**, **B**) One day later, BAL total and differential cell counts were determined and compared among WT, *mev*/+, *Kit^W-sh^*, and *mev*/+/*Kit^W-sh^* mice treated with LPS or PBS. *Kit^W-sh^* mice received adaptively transferred BMMC from either WT or *mev/mev* mice and stimulated with LPS were also shown (* *P* < 0.05; ** *P* < 0.01). (**C**). Mast cell reconstitution in *Kit^W-sh^* mice. Toluidine blue staining (40x) and mast cell counts in the spleen sections from *Kit^W-sh^* mice that received either WT or *mev/mev* BMMC as compared to WT and *Kit^W-sh^* controls. (**D**). Lung histology. H&E stained lung sections from WT, *mev/+*, *Kit^W-sh^*, and *mev/+/Kit^W-sh^* mice with or without LPS stimulation. Shown are representative slides of at least 5 individual samples for each group (Magnification 20x).

To assess airway inflammation we determined the BAL cellularity and lung histology 24 hrs after airway LPS stimulation. Since *mev/mev* mice develop spontaneous inflammation in the airways and heterozygous *mev*/+ mice are phenotypically normal, *mev*/+ mice and WT mice were tested and compared in these studies. All mice in PBS control groups had baseline cell counts in the BAL fluid. Upon LPS stimulation, WT mice showed increased BAL cell counts, consistent with our previous observations [7]. However, *mev/+* mice had further increased BAL cell counts compared to WT mice (**Figure 2A**). Differential cell counts showed that WT mice had increased macrophages and neutrophils whereas the increase in eosinophils did not reach statistical significance compared to the PBS group. On the other hand, *mev/+* mice had further increased macrophages, neutrophils and eosinophils compared with WT mice (**Figure 2B**). Interestingly, with LPS stimulation, neither total cell counts nor differential cell counts were significantly increased in mast cell-deficient *Kit^W-sh^* mice (*mev/+/Kit^W-sh^* and *Kit^W-sh^*). However, when bone marrow derived mast cells (BMMC) from WT or *mev/mev* mice were transferred to *Kit^W-sh^* mice, the inflammatory responses were restored (**Figure 2A**). WT and *mev/mev* BMMC expanded in the spleen of *Kit^W-sh^* recipient mice in a similar manner (**Figure 2C**). These results suggest that mast cells play a critical role in LPS induced airway inflammation. Lung histology findings were consistent with those of BAL cellularity described above (**Figure 2D**).

**Figure 3:**
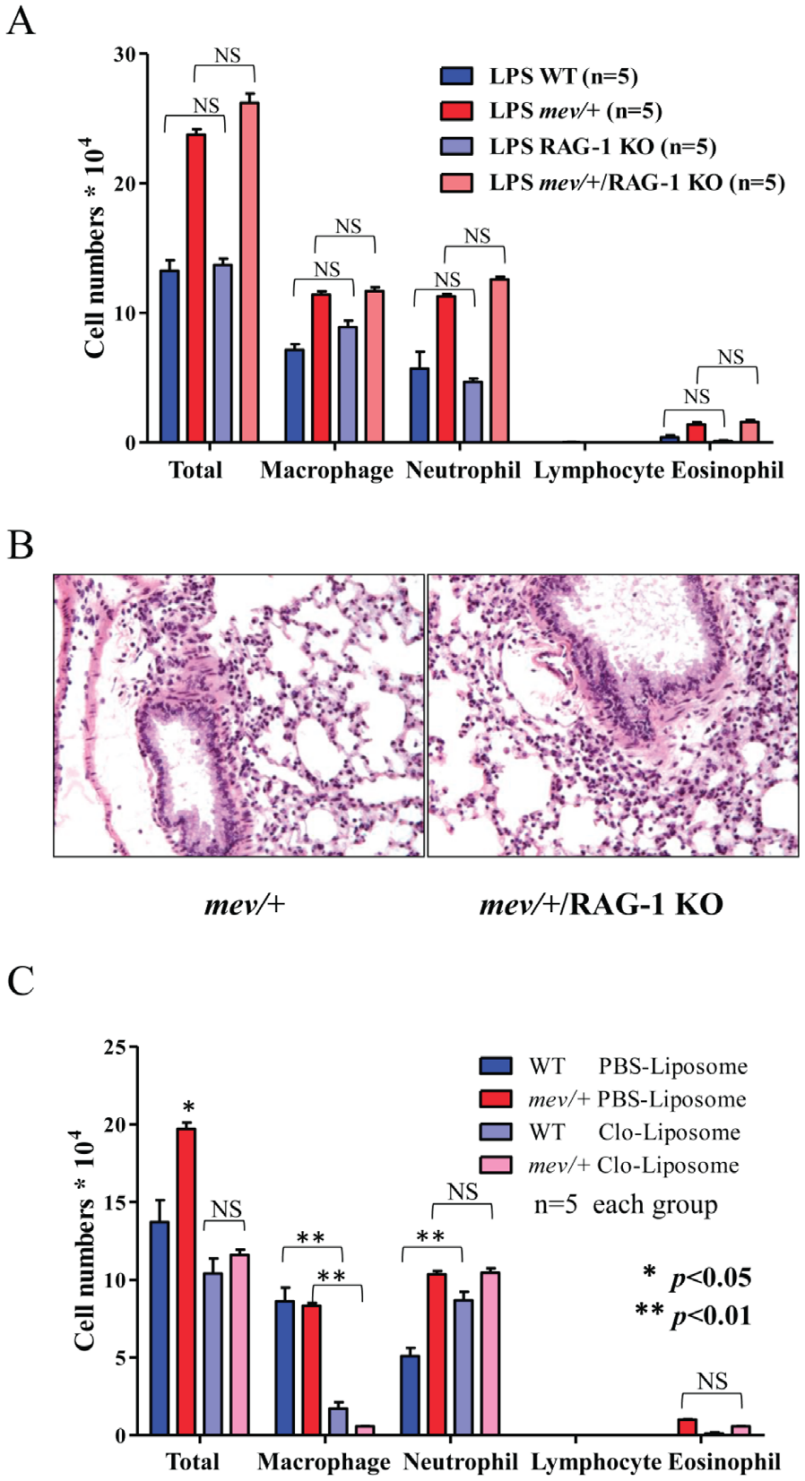
Lack of involvement of T cells and B cells in LPS induced airway inflammation. BAL total and differential cell counts (**A**) were determined and compared between LPS stimulated RAG-1 KO and WT mice; *mev*/+/RAG-1 KO and *mev*/+ mice (NS  =  not significant). (**B**). Lung histology of LPS stimulated *mev*/+ and *mev*/+/RAG-1 KO mice (Magnification 20x). (**C**). Effects of alveolar macrophage depletion on LPS induced airway inflammation and SHP-1 regulation. WT and *mev*/+ mice received airway administration (i.t.) of Clodronate (Clo)-Liposome or PBS-Liposome 24 hrs prior to LPS stimulation. BAL total and differential cell counts were determined and compared after LPS stimulation as described above (n = 5 for each group; * *P*<0.05; ** *P*<0.01).

To determine whether T cells or B cells are involved in LPS stimulated airway inflammation, we tested *mev/+* mice on RAG-1 null background since these mice do not have functional T or B cells. The results showed that mice with RAG-1 null mutation with or without SHP-1 deficiency had similar responses in total cell counts, differential and lung histology (**Figure 3A, 3B**). To test if alveolar macrophages are involved, we did the same experiments but with alveolar macrophage depletion by intratracheal instillation of Clodronate-liposome. PBS-liposome control did not have any effect on LPS stimulated airway inflammation, whereas Clodronate-liposome reduced the total cell counts in both WT and *mev/+* mice (**Figure 3C**). The change was mainly from reduction in macrophages, consistent with the expected effect of Clodronate-liposome. It was noticed that neutrophils in the WT mice given Clodronate-liposome were significantly more than those in WT mice that received PBS-liposome. This suggests that alveolar macrophages may have suppressive effect on inflammation [28]. Further increase in neutrophils was not seen in mev/+ mice that received Clodronate-liposome. Lymphocytes and eosinophils were not affected (**Figure 3C**). These data suggest that T cells, B cells and alveolar macrophages are not involved in LPS stimulated airway inflammation.

### SHP-1 in IgE-Mediated PSA and OVA Induced ASA

**Figure 4:**
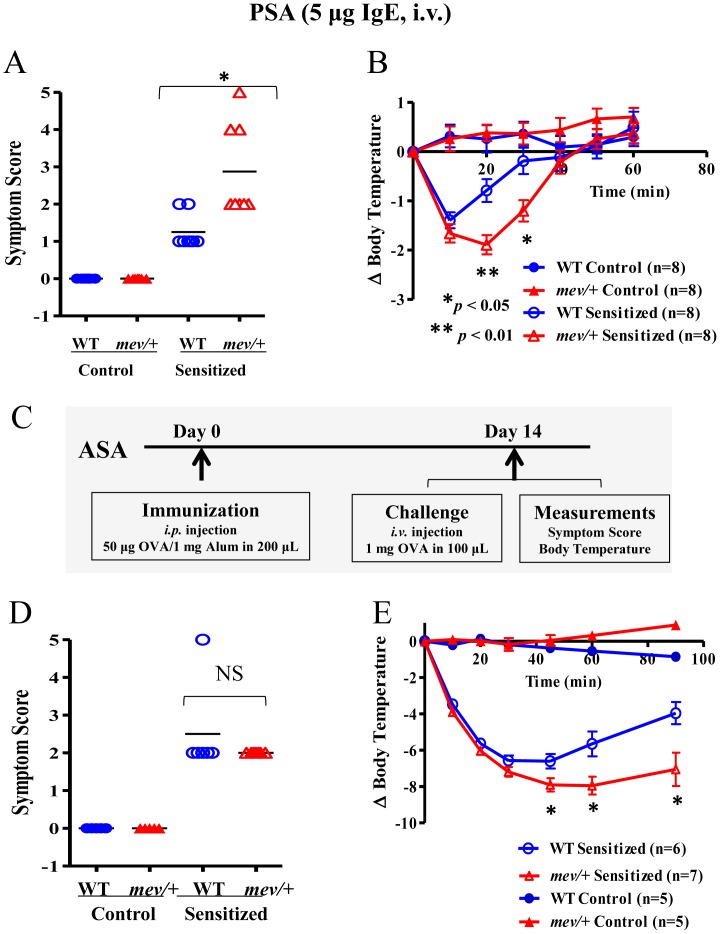
SHP-1 in IgE-mediated passive systemic anaphylaxis (PSA) and in OVA induced active systemic anaphylaxis (ASA). In PSA, WT and *mev/+* mice were passively sensitized (i.v.) with monoclonal anti-DNP IgE (5 µg) or PBS control. All mice were challenged with 1 mg DNP-HSA (i.v.) 24 hrs later. (**A**). Clinical symptom scores and (**B**). Body temperature changes as recorded at time intervals indicated (n = 8 for each group). For ASA, (**C**). Experimental protocol for OVA allergen induced ASA. (**D**). Anaphylactic clinical symptom scores and (**E**). Core body temperature changes were recorded (n = 5–7 for each group; NS  =  not significant; * *P*<0.05, ** *P*<0.01).

To understand SHP-1 regulation of IgE-dependent anaphylaxis, we tested *mev/+* mice using the PSA model. When challenged with 2,4-Dinitrophenyl hapten conjugated to human serum albumin (DNP-HSA), control mice without anti-DNP IgE sensitization did not manifest any anaphylactic reactions (**Figures 4A, 4B**). IgE-sensitized WT mice developed clinical scores of 1-2, with muzzle scratching and ear canal digging with hind legs, and some progressing to puffiness around mouth and self-isolation. On the other hand, IgE-sensitized *mev*/+ mice showed more severe responses, including prolonged periods of motionless, lack of response to whisker stimulation or prodding, and some to loss of consciousness and death (**Figure 4A**). In addition, changes in the core body temperature were closed correlated with the symptom scores (*P*<0.01; **Figure 4B**).

To determine whether SHP-1 plays any role in the development of active systemic anaphylaxis, which includes both sensitization and effector phases, we tested *mev/+* mice in the OVA-induced ASA model (**Figure 4C**). Control mice did not manifest any clinical responses or body temperature change (**Figure 4D, 4E**). In OVA sensitized groups, both WT and *mev/+* mice showed anaphylactic reactions to OVA challenge. Although there was no significant difference between the two groups in the clinical scores (**Figure 4D**), the reduction in body temperature in OVA-sensitized *mev/+* mice was significantly more severe than that of WT mice (**Figure 4E**). In addition, it was noticed that the recovery of body temperature of *mev/+* mice was much slower than that of WT mice (**Figure 4E**). These results suggest that SHP-1 deficiency has some effects on the core body temperature response but not on the clinical symptoms.

### OVA-specific IgE, IgG1 and IgG2a Production

**Figure 5:**
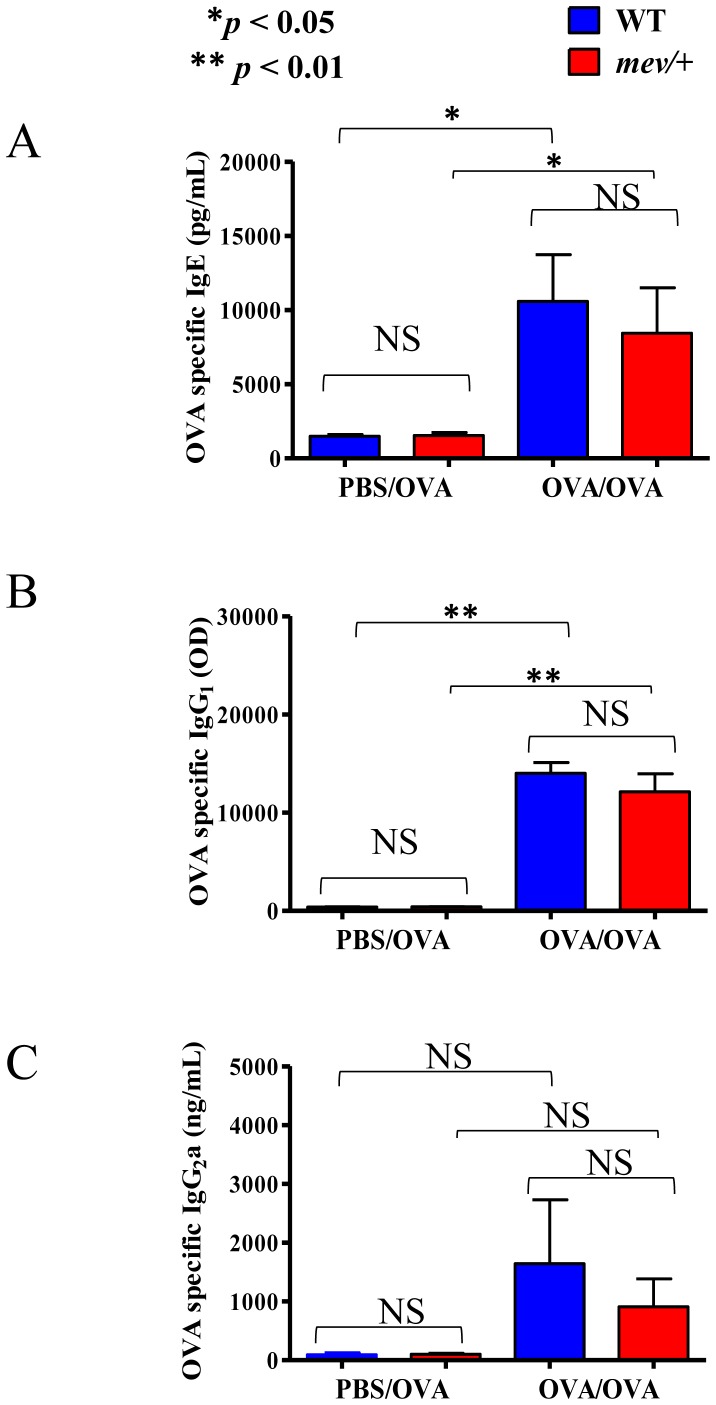
Serum levels of OVA-specific immunoglobulins. Serum samples of WT and *mev/+* mice were collected within 2 hrs after challenge and OVA allergen-specific IgE, IgG1 and IgG2a were measured by ELISA. (**A**). Serum OVA-specific IgE. (**B**). Serum OVA-specific IgG1. (**C**). Serum OVA-specific IgG2a. (n = 5–7 mice each group; * *P*<0.05; ** *P*<0.01).

To determine if SHP-1 regulates immunoglobulin responses in the ASA model, we collected blood samples from the mice and measured the serum levels of OVA-specific IgE, IgG1 and IgG2a by ELISA. Unsensitized mice had only background levels of OVA specific-IgE, IgG1 and IgG2a. On the other hand, significant amounts of OVA antigen-specific IgE (**Figure 5A**) and IgG1 (**Figure 5B**) were detected in the serum samples from OVA-sensitized and challenged WT and *mev/+* mice. However, there was no significant difference between WT and *mev/+* mice (**Figure 5A, 5B**). There was some increase in the serum levels of OVA-IgG2a in WT and *mev/+* mice, but the changes did not reach statistical significance compared to that of unsensitized WT or *mev/+* mice (**Figure 5C**). These results suggest that SHP-1 deficiency had no effect on the generation of immunoglobulin in this ASA model.

### SHP-1 Regulation of Anaphylactic Responses through Mast Cells

**Figure 6:**
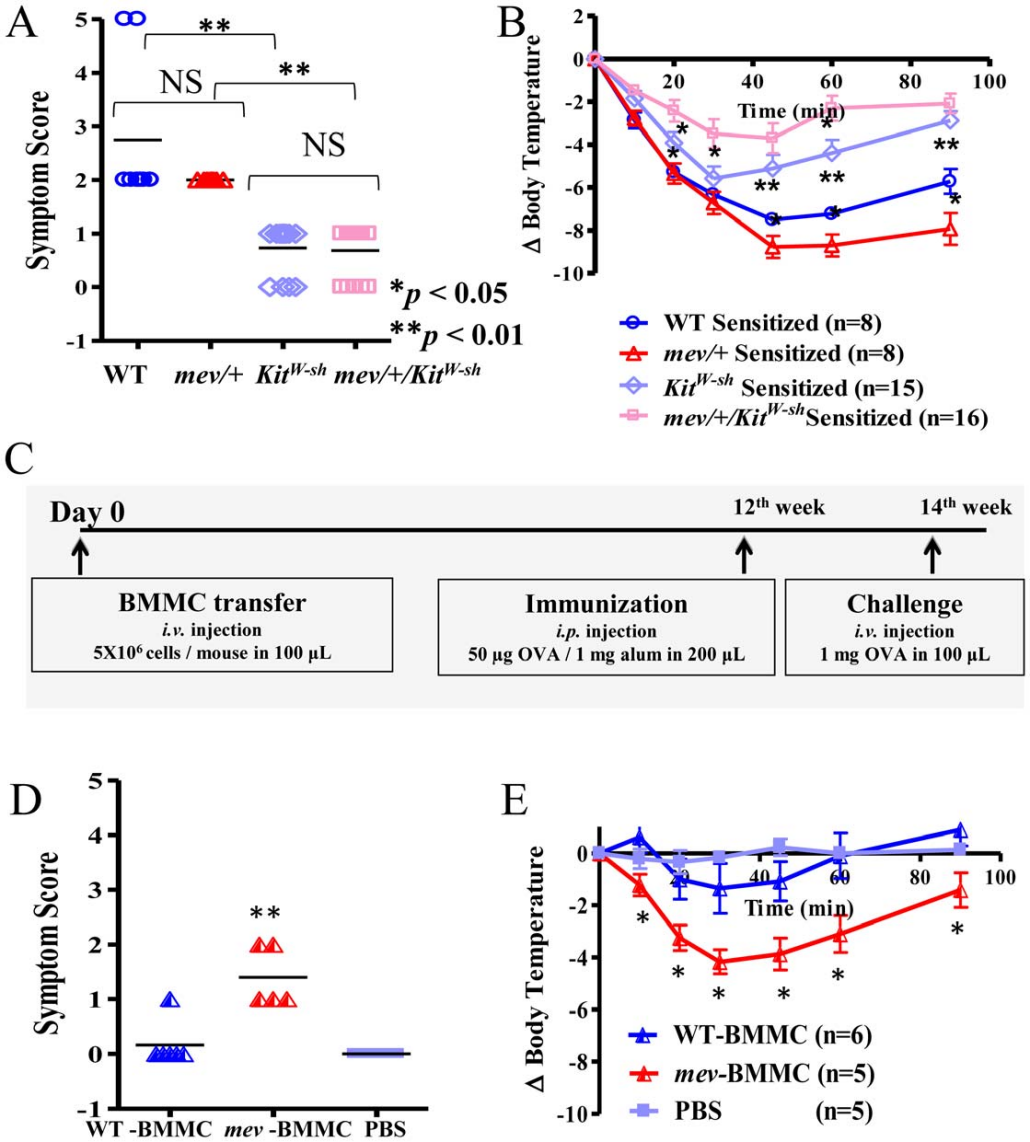
Role of mast cells in OVA induced ASA. WT, *mev/+*, *Kit^W-sh^*, and *mev*/+/*Kit^W-sh^* mice were sensitized and challenged. (**A**). Anaphylactic symptom scores and (**B**). Core body temperature changes were recorded. (**C**). Experimental protocol for BMMC transfer into mast cell deficient *Kit^W-sh^* mice and OVA allergen induced ASA in recipients. (**D**). Anaphylactic symptom scores and (**E**). Core body temperature changes. (NS  =  not significant; * *P*<0.05 and ** *P*<0.01).

We next determined whether SHP-1 regulation of anaphylactic responses was through mast cells. Mast cell-deficient *Kit^W-sh^* and *mev/+/Kit^W-sh^* mice were evaluated and compared to WT and *mev/+* mice in the OVA induced ASA model. Control unsensitized *Kit^W-sh^* and *mev*/+/*Kit^W-sh^* mice did not show any response to OVA challenge (data not shown). Compared to WT and *mev/+* mice, sensitized *Kit^W-sh^* or *mev*/+/*Kit^W-sh^* mice developed significantly reduced clinical scores (**Figure 6A**). Also, *Kit^W-sh^* mice had significantly less body temperature reduction compared to WT mice. Similarly, *mev/+/Kit^W-sh^* mice had reduced temperature change compared to *mev*/+ mice (**Figure 6B**). These observations indicate that mast cells are important in clinical response and in body temperature change in anaphylaxis. Surprisingly, SHP-1 deficient *mev/+/Kit^W-sh^* mice showed significantly less reduction in body temperature compared with *Kit^W-sh^* mice (**Figure 6B**), suggesting that SHP-1 may have some effect on factors other than mast cells that also contribute to this response. To further determine the role of mast cells and SHP-1 in the ASA responses BMMC from WT and *mev/mev* mice were adoptively transferred to mast cell deficient *Kit^W-sh^* mice and the recipients were examined in the ASA model. The results showed that *Kit^W-sh^* mice received WT mast cells (WT-BMMC) had mild temperature changes and clinical scores that did not reach statistical significance below baseline (PBS). However, *Kit^W-sh^* mice received *mev/mev* mast cells (*mev*-BMMC) had significant temperature reduction and clinical symptoms at every time point examined (**Figure 6D, 6E**). Taken together, these results demonstrated that mast cells are essential in allergen induced anaphylaxis and SHP-1 is a critical factor in this process through regulation of mast cell functions.

### SHP-1 in Basophil Differentiation and Growth

**Figure 7:**
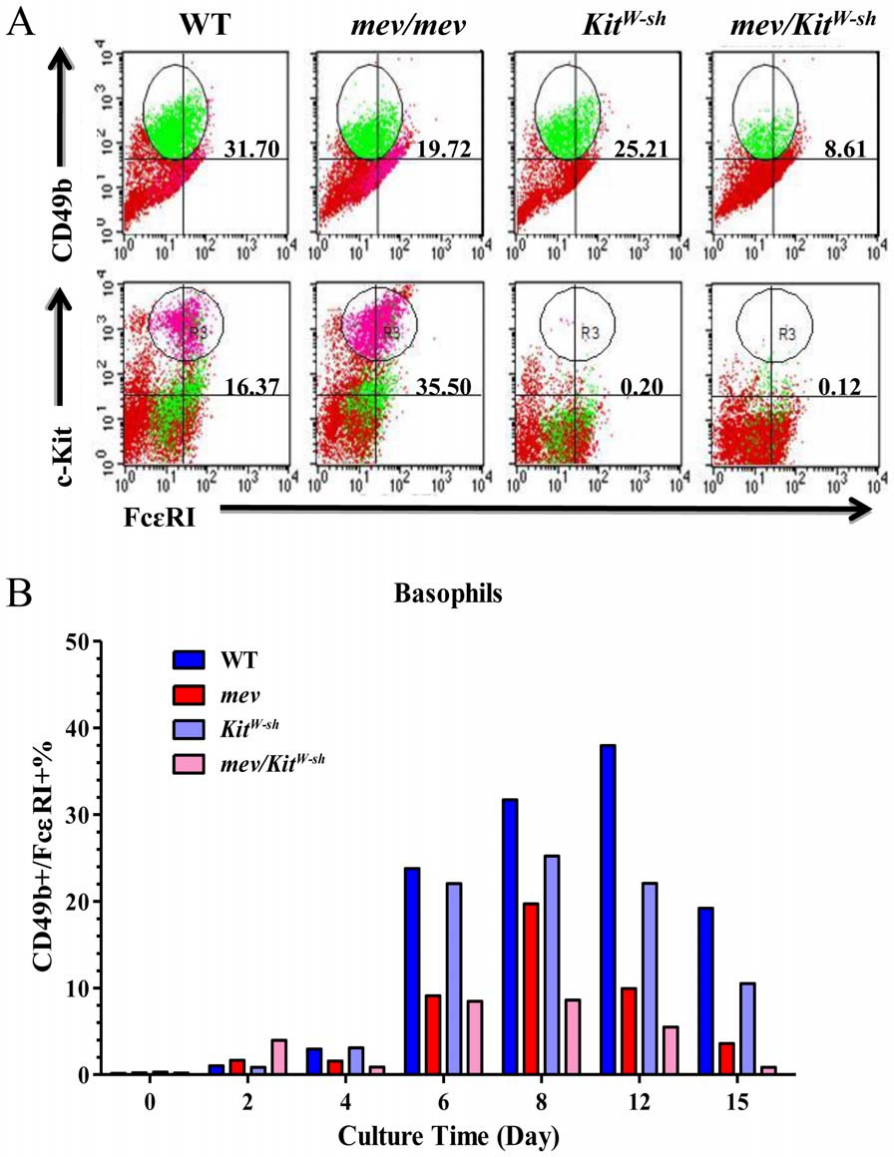
SHP-1 regulation of basophil differentiation. Bone marrow cells from WT, *mev/mev*, *Kit^W-sh^*, and *mev*/*Kit^W-sh^* mice were cultured in IL-3-containing medium for specified periods of time and the percentage of mature mast cells and basophils were determined. Basophils and mast cells were identified by surface markers CD49b and c-Kit, respectively, in addition to FcεRI by FACS. (**A**). Percentage of mature basophils and mast cells in different groups on day 8. (**B**). Kinetics of basophil differentiation from day 0 to day 15. Shown is a representative of three independent experiments with similar results.

We next evaluated whether SHP-1 has any regulatory function in basophil differentiation and maturation by determining the percentage of mature basophils using FACS analysis of cell surface markers CD49b and FcεRI at different time points in culture. Shown in **Figure 7A**, on day 8 of culture in IL-3-containing medium, as expected, no c-Kit/FcεRI positive mast cells were found in bone marrow culture of *Kit^W-sh^* or *mev*/*Kit^W-sh^* mice. The percentage of mast cells from *mev/mev* mice was significantly higher than that of WT mice, consistent with our previous report [27]. In contrast, the percentage of CD49b/FcεRI positive mature basophils in *mev/mev* bone marrow culture was significantly lower than that in WT bone marrow culture from day 8 (**Figure 7A**) as well as from day 4 to day 15 (**Figure 7B**). It was also noticed that c-Kit deficiency in *Kit^W-sh^* or *mev*/*Kit^W-sh^* bone marrow cells caused a reduction in the percentage of mature basophils in both groups with a similar pattern as that of WT and *mev/mev* groups (**Figure 7A, 7B**). These data suggest a critical role of SHP-1 in basophil differentiation and maturation as a positive regulator and c-Kit also plays an important role in the process.

## Discussion

SHP-1 has been shown as a critical regulator in pulmonary immunological homeostasis and in the pathogenesis of allergic immune responses, including spontaneous pulmonary inflammation, mucous hyperproduction, allergen induced immune and inflammatory responses, and in mast cell differentiation and function [23], [24], [25], [26], [27]. However, the role of SHP-1 in allergic anaphylactic responses is not known. In this study we tested the hypothesis that SHP-1, through regulation of mast cell function, is an important regulator of LPS induced airway inflammation and allergen induced anaphylactic responses.

LPS induced low-grade inflammation is an important part in the induction of adaptive Th2 immunity through the airway [6], [7], [8]. However, whether other innate immune cells are also involved in this process is not clear. Mast cells express TLR-4 and are able to respond to LPS stimulation by producing different cytokines, including TNF-α and IL-13 [27], [29], [30], [31], [32]. Based on our previous observation on SHP-1 regulation of mast cell function, we explored the interaction between SHP-1 and mast cells in allergic inflammation and anaphylactic responses *in vivo*.

We first determined whether mast cells can derive from tissues other than bone marrow in SHP-1 deficiency, which could be a potential mechanism for increased mast cells and mast cell mediated responses in various tissues seen in *mev/mev* mice [27]. Indeed, in SHP-1 deficiency substantial mast cells can grow from lung tissues and, surprisingly, mast cells develop from spleen with high efficiency. These are in contrast to SHP-1 normal tissues that are poor sources of mast cells compared to bone marrow. In fact, no mast cells were derived from lung tissues of WT mice (**Figure 1**). These findings suggest that given sufficient growth factors IL-3 and SCF, mature mast cells can grow in not only bone marrow but also extramedullary tissues that are deficient in SHP-1. These are consistent with our previous observations of increased mast cells and mediators in tissues [27]. Examination of heterozygous *mev/+* mice also showed increased mast cell mediators in spleen and lung tissues compared to those from WT mice (data not shown). These could be the cellular basis for enhanced allergic and inflammatory responses in these mice.

Next we investigated the involvement of SHP-1 and mast cells in LPS induced airway inflammation which could be an important part of allergen induced adaptive immune response. SHP-1 deficiency led to increased inflammation in the airway (**Figure 2**). More importantly, LPS induced airway inflammation is dependent on the presence of mast cells, since mast cell deficient *Kit^W-sh^* mice could not mount an inflammatory response to LPS, regardless of SHP-1 status. However, the response in *Kit^W-sh^* mice could be restored by adoptive transfer of mast cells. On the other hand, T cells or B cells do not seem to be involved in this process. Interestingly, alveolar macrophages appear to have some protective effect in WT mice since depletion of macrophages by Clodronate-liposome slightly but significantly increased LPS induced neutrophils in the airway (**Figure 3**). However, this was not evident in *mev/+* mice.

The results in the PSA model indicate that SHP-1 is a critical regulator in the response phase, possibly through regulation of mast cells (**Figure 4**). Further studies using *mev*/+ mice in the ASA model showed that SHP-1 deficiency resulted in increased anaphylactic responses in body temperature changes, consistent with those in the PSA. However, SHP-1 deficiency had no effect on the immunoglobulin production, indicating that SHP-1 regulates the effector phase but not the sensitization phase of anaphylaxis. To determine if the effect of SHP-1 is through regulation of mast cells, we used mast cell deficient *Kit^W-sh^* mice in the ASA experiments. Indeed, when *Kit^W-sh^* mice were tested, all anaphylactic responses, including clinical symptoms and core body temperature changes were significantly diminished. Furthermore, the anaphylactic responses in *Kit^W-sh^* mice were restored when reconstituted with mast cells derived from bone marrow of WT or *mev/mev* mice. These findings indicate that SHP-1 is critical in the effector phase of anaphylaxis through regulation of mast cells.

In these experiments we noticed that SHP-1 deficient and mast cell deficient *mev*/+/*Kit^W-sh^* mice had significantly less temperature decrease after allergen challenge compared to *Kit^W-sh^* mice, suggesting that besides mast cells SHP-1 may have effects on other cell types. Basophils also contribute to allergen induced anaphylactic responses [33], [34]. We investigated the potential effects of SHP-1 on basophils by examining basophil maturation in bone marrow cell culture. In contrast to the pattern of mast cell differentiation [27], the percentage of basophils derived from *mev/mev* bone morrow was significantly lower than that from WT bone marrow, suggesting a role of SHP-1 in basophil development. Reduced basophils in SHP-1 deficiency may explain the observation that *mev/+* mice rarely had extreme responses in ASA. In addition, the number of basophils derived from c-Kit deficient bone marrow was lower than that of WT bone marrow. It is known that basophil precursors, but not mature basophils, express c-Kit. These data indicate that c-Kit has some effect on basophil maturation, though not as essential as that for mast cells. The molecular mechanisms by which SHP-1 and c-Kit regulate basophil differentiation need to be further investigated.

These studies revealed that phosphatase SHP-1 is an important regulator in initiation of allergic airway inflammation and in anaphylactic responses to allergen. These effects are through regulation of mast cell development and functions in tissues. In addition, SHP-1 and c-Kit seem to be positive regulators for basophil differentiation and growth. These findings provide novel insights into the regulatory mechanisms that govern allergic inflammation and anaphylactic responses and will help us further understand allergic diseases.

## Materials and Methods

### Animals

Heterozygous and homozygous viable motheaten (*Ptpn6^me-v^*) mice (abbreviated as *mev/+* and *mev/mev*), mast cell-deficient (*Kit^W-sh^*), and RAG-1 knockout (RAG-1 KO) mice on C57BL/6 genetic background were purchased from the Jackson Laboratory (Bar Harbor, ME). Wild type (WT), *mev*/+, and *mev/mev* mice were bred as described [27]. To generate *mev/+* mice with mast cell deficiency, *mev/+* mice were crossbred to the *Kit^W-sh^* genetic background to obtain *mev/+/Kit^W-sh^* mice for experiments. For bone marrow cell culture, homozygous *mev/Kit^W-sh^* mice were generated in a similar fashion by crossbreeding *mev/mev* mice with *Kit^W-sh^* mice. The SHP-1 phosphatase enzyme activity in the lung tissues of *mev/+* and *mev/mev* mice is about 60% and 10%, respectively, relative to that of WT mice [24]. The genotype of the mice was determined following the protocols provided by the Jackson Laboratory. Mice were used for experiments at 7–9 weeks of age. All experiments involving mice were approved by the Johns Hopkins University IACUC.

### Culture and Analysis of Tissue-Derived Mast Cells and Basophils

To determine tissue-derived mast cells, single-cell suspensions were prepared from bone marrow (BM), spleen and lung of WT and *mev/mev* mice. Cells were seeded at 4×10^5^/mL and cultured in IL-3-containing WEHI conditioned medium [27]. At specified time, total cell numbers were counted, cells were stained for surface markers FcεRI and c-Kit, and analyzed by flow cytometry [27]. In some experiments BMMC were transferred to *Kit^W-sh^* mice as illustrated in **Figure 6C**, similar to the protocol as reported previously [35]. Briefly, 5×10^6^ BMMC in 100 µl medium were injected through tail vein to *Kit^W-sh^* mice. Twelve weeks later, recipient mice were used in the airway inflammation and anaphylaxis experiments as described below. Bone marrow-derived basophils were cultured in the same IL-3-containing medium and analyzed by FACS using surface markers CD49b and FcεRI. Under this culture condition, mature basophils appeared within a week, peaked around day 12 and disappeared after day 15 (**Figure 7B**).

### LPS Induced Airway Inflammation

Mice were stimulated i.n. with 100 ng of LPS from *E. coli* O55:B5 (CalBiochem, San Diego, CA) for 24 hrs and BAL and lung samples were obtained as described previously [7], [36]. The total number of cells was determined after resuspending the cell pellet in 200 µL PBS. After cytospin and Diff-Quik staining (Dade Behring, Deerfield, IL), 500 cells were counted and differentiated. The lung was inflated and immersed in fixatives overnight before being processed for histology.

### Passive and Active Systemic Anaphylaxis (PSA and ASA)

To assess specific responses to IgE, mice were passively sensitized with 5 µg anti-DNP IgE mAb i.v. After 24 hrs, mice were challenged i.v. with 1 mg DNP-HSA antigen (Sigma-Aldrich, St. Louis, MO). Control mice received vehicle without IgE but were challenged with DNP-HSA. Anaphylactic responses, including core body temperature change (hypothermia) and clinical symptoms were determined. To induce ASA, mice were immunized and challenged as in Figure 4C.

### Assessment of Anaphylactic Responses

Immediately after i.v. injection of allergens, mice were monitored for 60 min in PSA and 90 min in ASA experiments for the development of clinical symptoms and core body temperature changes as described previously by Sun et al. [37]. Core body temperature was measured using a digital thermometer with a rectal probe attached to FlexiVent Ventilator (SCIREQ, Inc., Montreal, Canada).

### Measurement of OVA-Specific Immunoglobulins

Peripheral blood was collected at the end of OVA challenge in ASA. Serum levels of OVA-specific IgE, IgG1 and IgG2a were determined by ELISA as described previously [38]. The optical density of the reactions was measured by a microplate reader at 450 nm. Purified mouse IgE and IgG2a were used as standards (BD Pharmingen) and the levels of IgE and IgG2a were calculated accordingly. The IgG1 levels were expressed in O.D.

### Depletion of Alveolar Macrophages

For alveolar macrophage depletion, mice were given an intratracheal (i.t.) injection of 100 µL of Clodronate-liposome or PBS-liposome control one day before LPS stimulation. Clodronate was a gift of Roche Diagnostics GmbH (Mannheim, Germany) and was encapsulated in liposomes in the laboratory of Dr. Nico van Rooijen (Amsterdam, The Netherlands).

### Statistical Analysis

Data were assessed by *Student's t* test for comparison between two groups or by ANOVA for comparison among multiple groups. The results were expressed as Mean±SEM. Differences with P values less than 0.05 were considered statistically significant.
